# Pedigree analysis exploring the inconsistency between diverse phenotypes and testing criteria for germline *TP53* mutations in Chinese women with breast cancer

**DOI:** 10.1007/s10549-024-07341-7

**Published:** 2024-06-15

**Authors:** Xin Huang, Chang Chen, Yan Lin, Changjun Wang, Xingtong Zhou, Ying Xu, Qiang Sun, Yidong Zhou

**Affiliations:** Department of Breast Surgery, Peking Union Medical College Hospital, Beijing, China

**Keywords:** *TP53* mutation, Breast cancer, Pedigree analysis, De novo mutation, Mosaic mutation, Phenotype

## Abstract

**Purpose:**

In the present study, we addressed the inconsistency between the testing criteria and diverse phenotypes for germline *TP53* mutation in patients with breast cancer in the Chinese population.

**Method:**

We proposed a new added item (synchronous or metachronous bilateral breast cancer) as one of the testing criteria (aimed at high-penetrance breast cancer susceptibility genes) and applied it for determining *TP53* germline mutation status in 420 female patients with breast cancer using multigene panel-based next-generation sequencing, Sanger sequencing, and mass spectrometry.

**Results:**

We found that 1.4% of patients carried a pathogenic or likely pathogenic germline *TP53* mutation. Compared with *BRCA* mutation carriers (8.0%) and non-carriers (7.1%), *TP53* mutation carriers (33.3%) developed breast cancer earlier. The majority of *TP53* mutation carriers (66.7%) developed breast cancer after age 30 and had bilateral breast cancer (33.3%). Pedigree investigation of four *TP53* carriers and a patient with a *TP53* variant of unknown significance revealed that neither of their parents harbored the same mutations as the probands, indicating that the mutations might occur de novo.

**Conclusion:**

Our study revealed distinguishing features of *TP53* carriers among Chinese women with breast cancer, which is inconsistent with the currently used testing criteria; therefore, the newly proposed testing criteria may be more appropriate.

**Supplementary Information:**

The online version contains supplementary material available at 10.1007/s10549-024-07341-7.

## Introduction

Germline *TP*53 mutations are associated with Li-Fraumeni syndrome (LFS), a rare autosomal dominant inherited cancer predisposition disorder characterized by elevated cancer risk [1]. The most frequent LFS-associated ‘core’ cancers are osteosarcoma, soft-tissue sarcomas (STS), premenopausal breast cancer, and brain tumors, beginning in infancy [2, 3]. The reported cumulative cancer incidence among female *TP53* carriers is approximately 50% at age 30 and nearly 100% until age 70 [4]. Patients with *TP*53 germline mutations are characterized by more aggressive disease and worse overall survival [5]. Therefore, it is important to identify *TP53* carriers.

Previous studies have generally investigated germline *TP*53 mutations in affected individuals, such as women developing breast cancer before 31 years of age, individuals who met the criteria for LFS or Li-Fraumeni-like syndrome, or unaffected individuals from a family with a known *TP53* pathogenic or likely pathogenic (P/LP) variant [6, 7]. However, the extension of the scope from single-gene testing to multiplex genetic panel testing (MGPT), including *TP53*, has improved the detection rate of germline *TP53* variants in patients with breast cancer even if they do not fulfill the clinical testing criteria in China [3]. Therefore, *TP53* carriers have been identified in patients with breast cancer (not early_onset) or even in the proband in the absence of a typical family history of cancer. Sheng et al. reported that 50% of *TP53* carriers had no family history suggestive of any cancer [8]. They also observed a mean onset age of 43.1 years, contrary to the threshold for early_onset breast cancer in *TP53* carriers; *TP*53 mutation carriers were especially older at the initial diagnosis of breast cancer [9, 10]. Moreover, bilateral breast cancer is more common in *TP53* carriers; however, it is neither one of the Chompret criteria for LFS nor one of the testing criteria for germline *TP53* mutations [8]. These diverse phenotypes are inconsistent with previous testing criteria for patients with breast cancer with germline *TP53* mutations [11].

For women with breast cancer carrying germline *TP53* mutations, unnecessary radiation therapy (RT) is contraindicated [12]. Considering that the RT-associated cancer risk increases due to lack of restoration from tissue damage following DNA-damaging RT in *TP53* carriers, mastectomy is the recommended surgical management for such patients, whereas RT is necessary for breast-conserving surgery (BCS) [12–14]. If a patient with breast cancer carrying a germline *TP53* mutation is not identified because without typical family history or not early_onset breast cancer, inappropriate surgical management will bring severe adverse consequences.

The underidentification of patients with breast cancer may be related to the inconsistency between testing criteria for *TP53* carriers with breast cancer and diverse phenotypes in the Chinese population. As discussed, the previous testing criteria for *TP53* germline mutations are less accurate than expected. To address this issue, based on the testing criteria aimed at high-penetrance breast cancer susceptibility genes, we included other features as novel testing criteria for identifying *TP53* carriers with breast cancer and summarizing their characteristics [1]. More importantly, we investigated the pedigrees of *TP*53 mutation carriers to further explain why it is inconsistent between family history and *TP53* variant proband. The aim of the present study was to propose a novel approach of determining more suitable testing criteria for germline *TP53* mutations in Chinese breast cancer patients and to facilitate its implementation in clinical practice.

## Methods

### Ethics statement

The study was approved by the Ethics Committee of Peking Union Medical College Hospital (Approval No. ZS-1655) on July 24, 2018. Written informed consent was obtained from all participants [16]. The seven patients, presented as cases, provided written informed consent for the publication of any potentially identifiable data included in this article.

### Participants and samples

Based on the testing criteria aimed at high-penetrance breast cancer susceptibility genes from the National Comprehensive Cancer Network (NCCN) Guidelines (Supplementary Table 1), we added a new item to the testing criteria described in our previous study: (I) triple-negative breast cancer (TNBC) diagnosed at ≤ 60 years; (II) breast cancer diagnosed at ≤ 45 years; (III) breast cancer and at least one close blood relative with any cancer related to *BRCA1/2*; or (IV) synchronous or metachronous bilateral breast cancer [1, 17]. As a result, 432 Chinese women with primary invasive breast cancer, between 2016 and 2021, were retrospectively enrolled from Peking Union Medical College Hospital (Beijing, China) (Table 1). About 2–3 mL peripheral blood was obtained from all participants. They all underwent genetic testing. Subsequently, 12 patients were excluded owing to unqualified genomic DNA (gDNA) samples. Therefore, 420 patients were included and analyzed. Only *BRCA* and *TP53* mutations were determined; however, the clinical characteristics of all patients were obtained. Additionally, pedigree analysis was performed on the *TP53* probands and their family members. The personal information of the family members was also obtained.

**Table 1:** Clinical characteristics of breast cancer patients tested for germline mutations

Characteristics	Non-carriers^†^ No. (%) (*n* = 326)	*BRCA* mutation carriers No. (%) (*n* = 88)	*TP53* mutation carriers No. (%) (*n* = 6)
Mean age at first diagnosis, year (range) ≤ 30 > 30	45.6 (22–81) 23 (7.1) 303 (92.9)	40.7 (22–60) 7 (8.0) 81 (92.0)	32.5 (27–37) 2 (33.3) 4 (66.7)
Pathological type Invasive carcinoma Carcinoma in situ	310 (95.1) 16 (4.9)	86 (97.7) 2 (2.3)	6 (100.0) 0 (0)
Tumor grade I II III Unknown	77 (23.6) 126 (38.7) 107 (32.8) 16 (4.9)	1 (1.1) 32 (36.4) 43 (48.9) 12 (13.6)	1 (16.7) 3 (50.0) 2 (33.3) 0 (0)
Tumor stage 0 1 2 3 4	14 (4.3) 131 (40.2) 151 (46.3) 25 (7.7) 5 (1.5)	2 (2.3) 41 (46.6) 40 (45.5) 5 (5.7) 0 (0)	0 (0) 5 (83.3) 1 (16.7) 0 (0) 0 (0)
Lymph node stage 0 1 2 3	150 (46.0) 61 (18.7) 57 (17.5) 58 (17.8)	39 (44.3) 24 (27.3) 15 (17.0) 10 (11.4)	3 (50.0) 2 (33.3) 0 (0) 1 (16.7)
Hormone receptor status Positive Negative	122 (37.4) 204 (62.6)	40 (45.5) 48 (54.5)	3 (50.0) 3 (50.0)
HER2 status Positive Negative	23 (7.1) 303 (92.9)	3 (3.4) 85 (96.6)	2 (33.3) 4 (66.7)
Family history of breast cancer Yes No		54 (61.4) 34 (38.6)	2 (33.3) 4 (66.7)
Bilateral breast cancer Yes No	9 (2.8) 317 (97.2)	5 (5.7) 83 (94.3)	2 (33.3) 4 (66.7)

^†^: Here, non-carriers indicate that individuals with breast cancer carried neither germline *BRCA* nor *TP53* mutations

### DNA extraction, next-generation sequencing, Sanger sequencing, mass spectrometry, and quantitative polymerase chain reaction

Genomic DNA was extracted from peripheral mononuclear blood cells using the QIAamp DNA Blood Mini Kit (Qiagen, Hilden, Germany); the integrity, purity, and concentration of extracted DNA were detected and evaluated using agarose-gel electrophoresis, the NanoDrop2000 spectrophotometer, and the Qubit 2.0 fluorimeter (Thermo Fisher, USA) [15]. DNA with clear and non-dispersive bands on agarose-gel electrophoresis, an OD260/280 ≥ 1.8, a concentration ≥ 50 ng/µl, and a total content above 2 µg were sequenced [16]. DNA samples that do not meet the above quality criteria will result in inaccurate variant identification. Targeted DNA was amplified and sequenced by Beijing Genomics Institute on the MGISeq-2000 platform (MGI Tech Co., Shenzhen, China) with coverage depths of 500 − 900 × (MGI, Shenzhen, China) [17]. Clean data were aligned to the hg19 reference genome using GATK 4.0. (https://gatk.broadinstitute.org/hc/en-us/sections/360007407851-4-0-0-0). The details are outlined in our previous studies [17, 21]. Sanger sequencing in duplicate was performed to confirm all deleterious mutations. Unique variants were identified through mass spectrometry (Agena Bioscience, USA). The single-plex assay was designed using Assay Design Suite v2.0 (Agena Bioscience, USA) and synthesized by BGI Genomics (Beijing, China). Following standard procedures, 1 μL of genomic DNA was mixed with every 5 μL of the PCR reaction mixture. The data were collected using Mass ARRAY Typer v4.0 (Agena Bioscience). Large genomic rearrangements (LGR) were confirmed using quantitative polymerase chain reaction (qPCR) assays, as shown in Fig. 5.

### Variant interpretation

Variants were filtered to clear synonymous variants, germline variants in the Single Nucleotide Polymorphism database, and variants with a population frequency > 0.1% in the Exome Sequencing Project database [18]. The disease-causing potential of the variations was assessed based on the classification guidelines established by the American College of Medical Genetics and Genomics in 2015 [19]. In particular, the ClinGen TP53 Variant Curation Expert Panel was used for *TP53* germline mutation classification [20]. A positive family history was defined as the existence of close blood relatives who developed a history of LFS-associated and BRCA-associated ‘core’ cancers, such as breast cancer, ovarian cancer, STS, and brain tumor [1].

### Statistical analysis

This was a retrospective study with case presentations. Categorical variables were analyzed using Pearson’s χ2 test or Fisher’s exact test, and continuous variables were analyzed using Student’s t test or the Mann–Whitney U test to compare differences in clinicopathological characteristics among patients with *TP53* germline mutations, patients with *BRCA* germline mutations, and noncarriers, as described in our previous study [21].

## Results

### Retrospective assessment of TP53 germline mutations in patients with breast cancer

Eighty-eight patients (21.0%, 88/420) were found to carry a likely pathogenic or pathogenic mutation in *BRCA1* or *BRCA2,* among whom only one carried both *BRCA1* likely pathogenic and *BRCA2* pathogenic mutation. Six patients carried a *TP53* likely pathogenic/pathogenic mutation (1.4%, 6/420) (Case 1–6, Table 2). The clinicopathological characteristics of the 420 participants are shown in Table 1. The mean age of breast cancer onset in *TP53* mutation carriers tended to be lower than that in *BRCA* mutation carriers and non-carriers (who did not carry *BRCA* or *TP53* mutations) (mean age [range] in years: 32.5 [27–37] vs. 40.7 [22–60] vs. 45.6 [22–81], respectively). Compared to 8.0% (7/88) in *BRCA* mutation carriers and 7.1% (23/326) in non-carriers, although 33.3% (2/6) of *TP53* mutation carriers developed breast cancer before age 30 years, more (66.7%, 4/6) *TP53* carriers developed breast cancer after the age of 30 years. It seemed that *TP53* mutation carriers tended to develop HER2-positive breast cancer (33.3%, 2/6) more often than did *BRCA* mutation carriers (3.4%, 3/88) and non-carriers (7.1% (23/326). Notably, five (5.7%, 5/88) *BRCA* carriers and two (33.3%, 2/6) *TP53* carriers developed bilateral breast cancer. The majority of *BRCA* carriers (61.4%, 54/88) had a family history of breast cancer, whereas only two *TP53* carriers (33.3%, 2/6) did (Table 1). However, only two of six *TP53* mutation carriers had a family history suggestive of LFS (Table 2). One patient was found to have a *TP53* VUS, but the clinical manifestation conformed to a *TP53* deleterious mutation. The details of the patients with *TP53* variants, including six with deleterious mutations and one with VUS, are shown in Table 2. We investigated the pedigrees of five patients, including four *TP*53 carriers (Case 1–4) and one *TP53* VUS (Case 7). Unfortunately, pedigrees could not be investigated for Case 5 and Case 6, as blood samples could not be obtained. Surprisingly, neither the paternal nor maternal sides harbored the same variants as did the probands, both in Case 1–4 and Case 7.

**Table 2:** Specific characteristics of patients with *TP53* mutations

Case	*TP53* mutation	Mutation site	Amino acid alterations	Function change	VAF	Age at diagnosis	Hormone receptor status	HER2 status	Lymph node status	Family history suggestive of LFS	Sanger sequencing for the patient’s mother	Sanger sequencing for the patient’s father	Bilateral breast cancer
1	pathogenic	c.216_217insC	p.Val73Argfs*76	frameshift	29.3%	37	negative	positive	3/23	No	wild-type	wild-type	yes
2	likely pathogenic	c.711G > A	p.Met237Ile	missense	49.8%	30	negative	negative	1/25	No	wild-type	wild-type	yes
3	likely pathogenic	c.459delC	p.Gly154Alafs*16	frameshift	16.4%	33	negative	positive	negative	Yes	wild-type	wild-type	no
4	likely pathogenic	c.817C > T	p.Arg273Cys	missense	41.6%	27	positive	negative	negative	No	wild-type	wild-type	no
5	pathogenic	c.994-1G > T	NA	missense	45.28%	32	positive	negative	negative	No	NA	NA	no
6	pathogenic	c.375G > A	NA^†^	splice	49.29%	35	positive	negative	12/17	Yes	NA	NA	no
7	variant of uncertain significance	EX2_6 Dup	NA	duplication	NA	26	negative	negative	negative	No	wild-type	wild-type	yes

*LFS* Li-Fraumeni syndrome, *VAF* Variant Allele Frequency, *NA* not applicable

^†^: This patient had a shear region mutation that caused a similar frameshift mutation, and the termination codon was prematurely introduced

### Case presentations

**Fig. 1:**
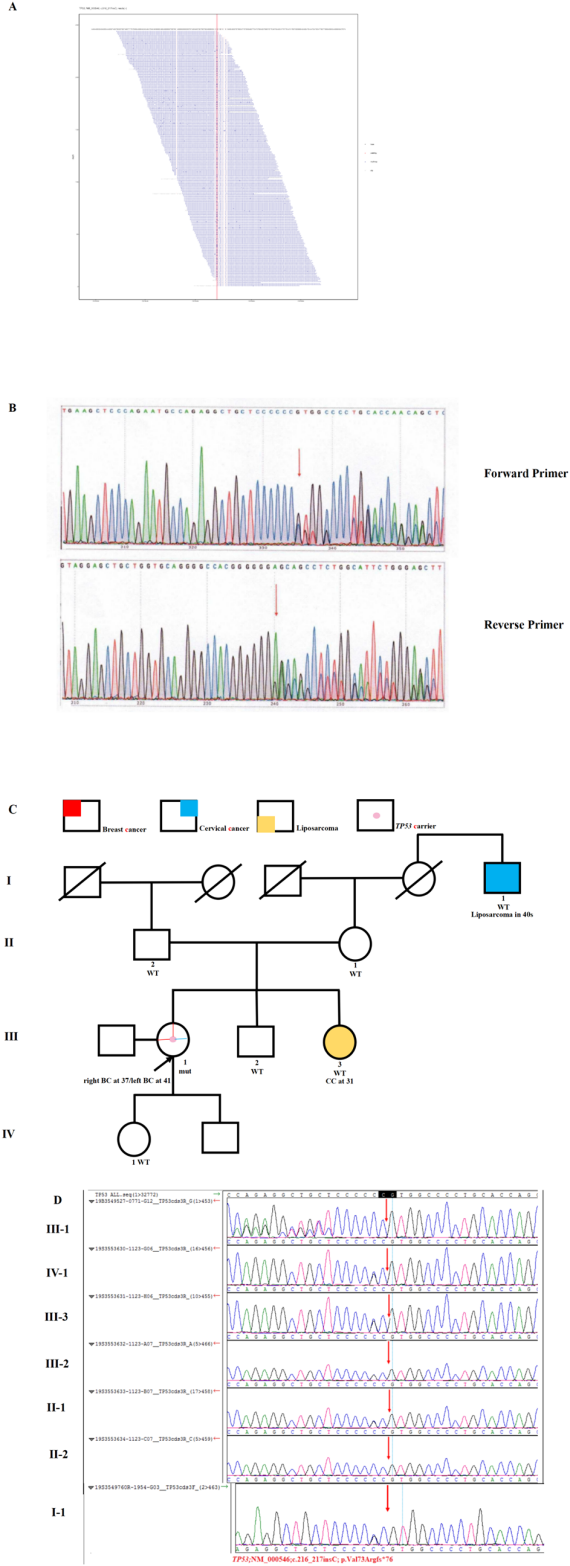
Germline *TP53* mutation identified in Case 1. **A** Sequencing data showing the *TP53* variant (NM_000546, c.216_217insC, p.Val73Argfs*76). **B** Pedigree of the family in Case 1. The proband is indicated with an arrow. A pink dot denotes a *TP53* carrier. The red color denotes the manifestation of breast cancer (BC); blue color, cervical cancer (CC); and yellow colour, liposarcoma. **C** Sanger sequencing of the *TP53* c.216_217insC mutation in the family showed that nobody harboured the variant. **D** PCR-Sanger confirmed the variant c.216_217insC as pathogenic via both the Forward Primer and Reverse Primer. The red arrows indicate the presence of a mutation. mut, mutant; WT, wild-type

Case 1 was a 41-year-old female, developing bilateral metachronous breast cancer at age 37 and 41 respectively. Similar pathology was observed in bilateral breast cancer, which showed invasive ductal carcinoma (IDC), categorized as estrogen receptor (ER)-negative, progesterone receptor (PR)-negative, and HER2-positive, with three involved lymph nodes in the right axilla and none in the left axilla. She had a history of cervical intraepithelial neoplasia (CIN III) and cervical conization. Her sister suffered from cervical cancer at age 31. Her grandmother’s brother developed pelvic liposarcoma in his 40 s. A germline frameshift mutation in *TP*53 (NM_000546.5: c.216_217insC, p.Val73fs), which can cause gene product deficiency, was detected in her blood sample (Fig. 1A and B) [22]. Therefore, it was classified as ‘pathogenic’ according to the ClinGen TP53 Variant Curation Expert Panel guidelines [20]. The variant allele frequency (VAF) was 29.3%. The patient’s pedigree is shown in Fig. 1C. Because of this finding, the patient’s parents, siblings, and daughter were tested for the *TP*53 c.216_217insC mutation; however, none harbored this variant (Fig. 1D).

**Fig. 2:**
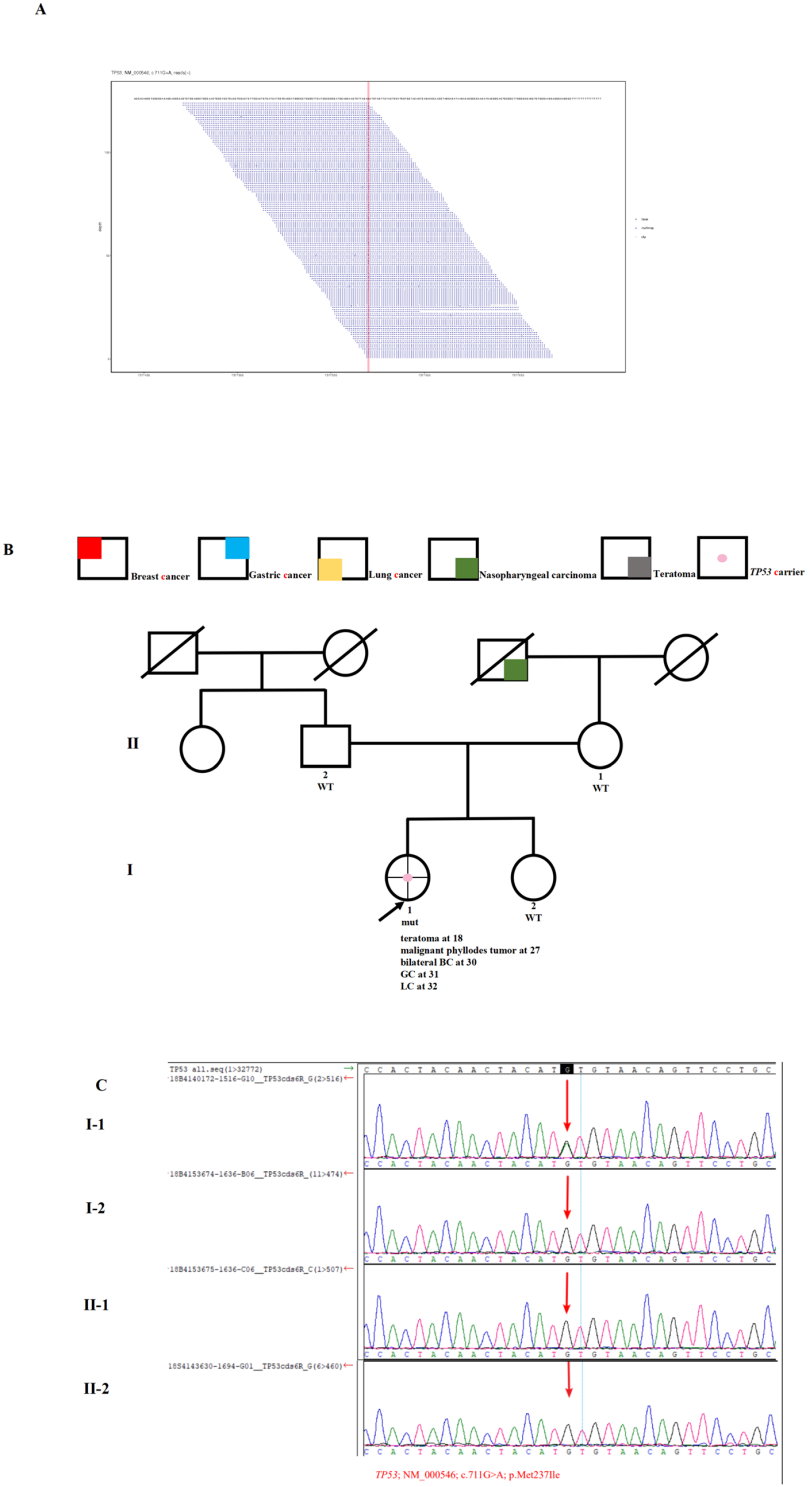
Germline *TP53* mutation identified in Case 2. **A** Sequencing data showing the *TP53* variant (NM_000546, c.711G > A, p.Met237Ile). **B** Pedigree of the family in Case 2. The proband is indicated with an arrow. The pink dot denotes a *TP53* carrier. The red colour denotes the manifestation of breast cancer (BC); blue, gastric cancer (GC); yellow, lung cancer (LC); green, nasopharyngeal carcinoma; and grey, teratoma. **C** Sanger sequencing of the *TP53* c.711G > A mutation in the family showed that nobody harboured the variant. The red arrows indicate the presence of the mutation. mut, mutant; WT, wild-type

Case 2 was a 32-year-old woman who underwent right mastectomy with axillary LN dissection at age 30. Pathology revealed that the tumors were polycentric (intraductal carcinoma in situ and IDC), with one LN involvement categorized as ER-negative, PR-negative, and HER2-negative. Only half a year later, she was found to develop left primary breast cancer with ER-positive, PR-negative, and HER2-negative IDC but without LN involvement. Unfortunately, she quickly developed gastric adenocarcinoma half a year later at age 31 and pulmonary invasive adenocarcinoma at age 32. Notably, she had teratoma at the age of 18 and recurrent malignant breast phyllodes tumors (PTs) between age 27 and 28. Her aunt on her father’s side developed lung cancer at age 55. The grandfather on her mother’s side developed nasopharyngeal carcinoma in his 40 s. She was recommended to undergo NGS testing, and a *TP*53 missense variant was detected (NM_000546: c.711G > A, p.Met237Ile; VAF 49.8%; Fig. 2A). Reportedly, this missense mutation can cause the amino acid 237 of the protein encoded by this gene to change from methionine to isoleucine, resulting in a decrease in *TP*53 transcriptional activity [23]. This mutation was classified as ‘likely pathogenic.’ The pedigree is shown in Fig. 2B. Due to these findings, all parents and siblings of the patient were tested for the *TP*53 c.711G > A mutation; however, none of them harbored the variant (Fig. 2C).

**Fig. 3:**
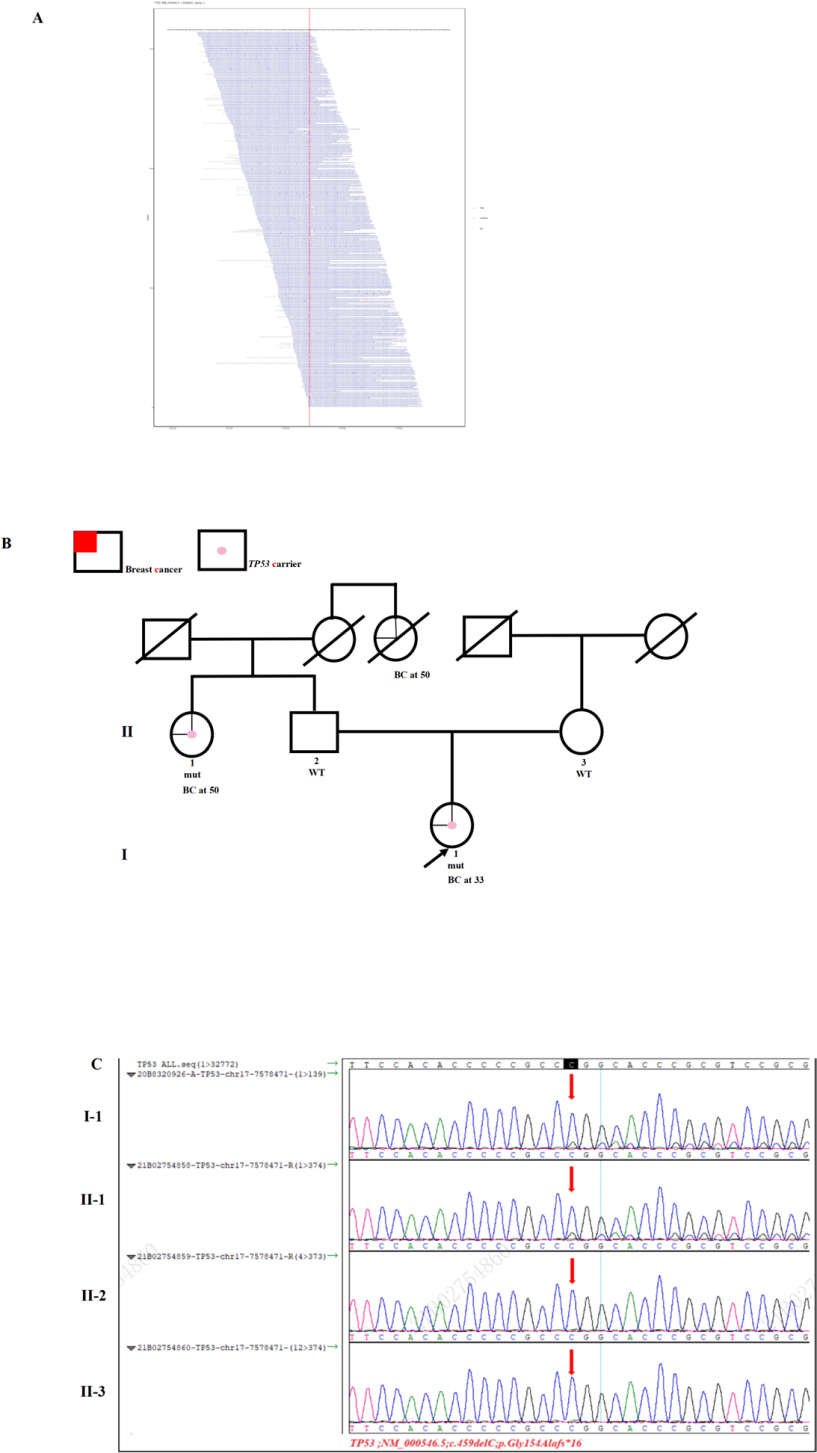
Germline *TP53* mutation identified in Case 3. **A** Sequencing data showing the *TP53* variant (NM_000546.5, c.459delC, p.Gly154Alafs*16). **B** Pedigree of the family in Case 3. The proband is indicated with an arrow. The pink spot denotes a *TP53* carrier. The red color denotes the manifestation of breast cancer (BC). **C** The *TP53* c.459delC mutation is not detected in her parents’ peripheral blood samples but detected in her aunt’s sample using Sanger sequencing. **D** Mass spectrometry confirms that the *TP53* c.459delC mutation is carried by both her and her aunt. mut, mutant; WT, wild-type. (Color figure online)

Case 3 was a 33-year-old female who developed unilateral breast cancer and underwent BCS and sentinel lymph node biopsy (SLNB). Pathology indicated ER-positive, PR-positive, and HER2-negative IDC. Her aunt on the father’s side had a history of breast cancer at age 50, so did her grandmother’s sister on her father’s side. NGS revealed that both the patient and her aunt on the father’s side carried a likely pathogenic *TP53* mutation (NM_000546.5; c.459delC, p.Gly154Alafs*16; VAF 16.4%; Fig. 3A). The pedigree is shown in Fig. 3B. This mutation was detected neither in her father’s nor in her mother’s peripheral blood using Sanger sequencing (Fig. 3C). Interestingly, the mutation failed to be detected via Sanger sequencing (sequencing depth: W571/M101, read ratio: 15.03%) both for her and her aunt on the father’s side, but was successfully confirmed via mass spectrometry (Fig. 3D).

**Fig. 4:**
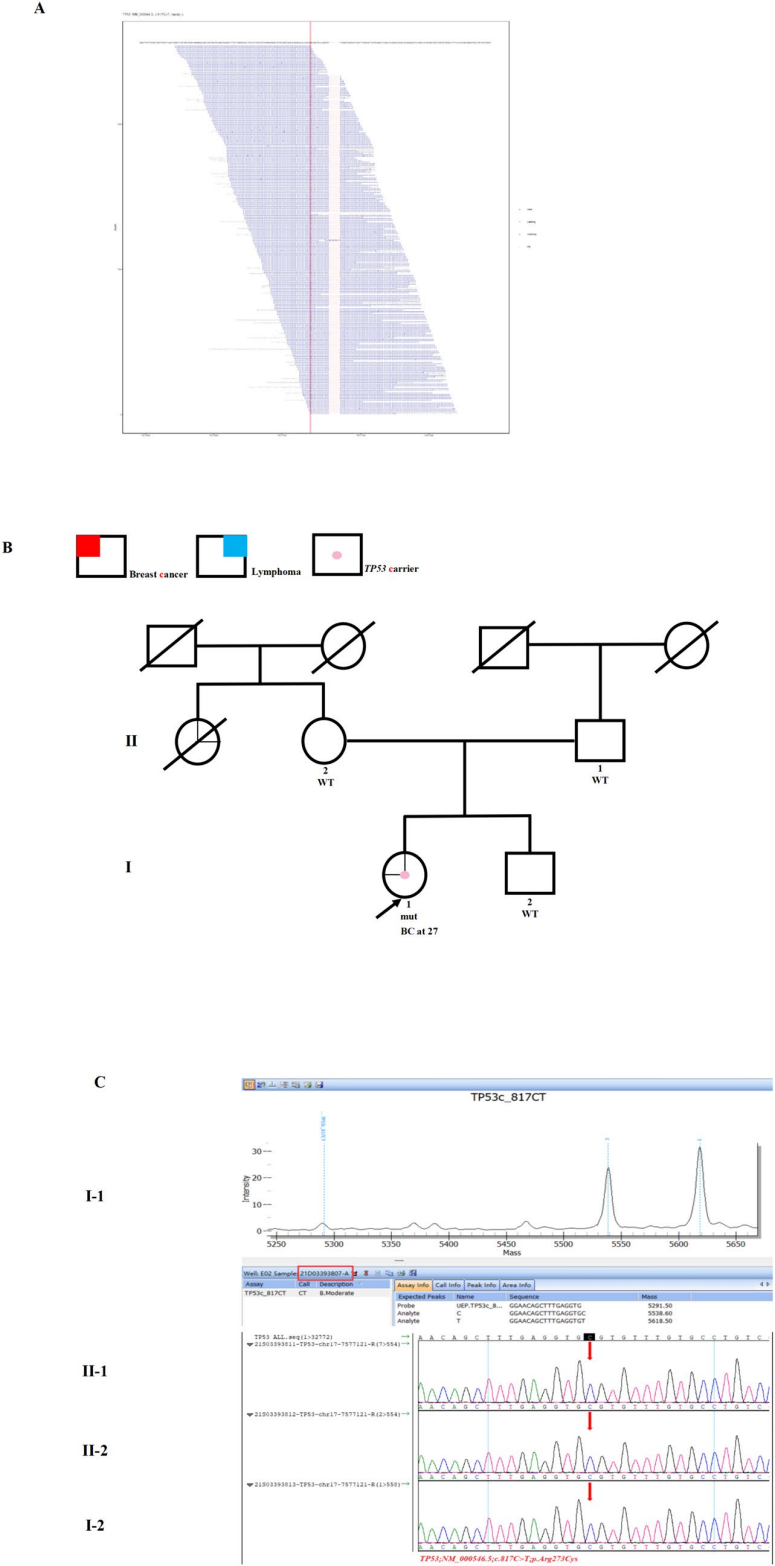
Germline *TP53* mutation identified in Case 4. **A** Sequencing data showing the *TP53* variant (NM_000546.5, c.817C > T, p.Arg273Cys). **B** Pedigree of the family in Case 4. The proband is indicated with an arrow. A pink dot denotes a *TP53* carrier. The red color denotes the manifestation of breast cancer (BC) and the blue color denotes lymphoma. **C** Mass spectrometry confirmed the presence of the *TP53* variant (NM_000546.5; c.817C > T), and Sanger sequencing showed that nobody harbored the variant in the family. The red arrows indicate the presence of the mutation. mut, mutant; WT: wild-type. (Color figure online)

Case 4 was a 28-year-old woman. Her right breast tumor was detected at age 27. Pathological examination showed invasive ER-positive, PR-positive, and HER2-negative IDC. Both BCS and SLNB were performed on this unmarried patient. After surgery, she underwent postoperative radiotherapy. Two years later, she was suspected of developing recurrent sarcoma on her right breast. She subsequently received MGPT, and a likely pathogenic *TP53* mutation was detected (NM000546.5; c.817C > T; VAF 41.6%; Fig. 4A). The missense mutation may cause the amino acid 273 of the encoded *TP53* protein to change from arginine to cysteine. Only her aunt on her mother’s side had a history of lymphoma (Fig. 4B). None of the relatives harbored this variant (Fig. 4C).

**Fig. 5:**
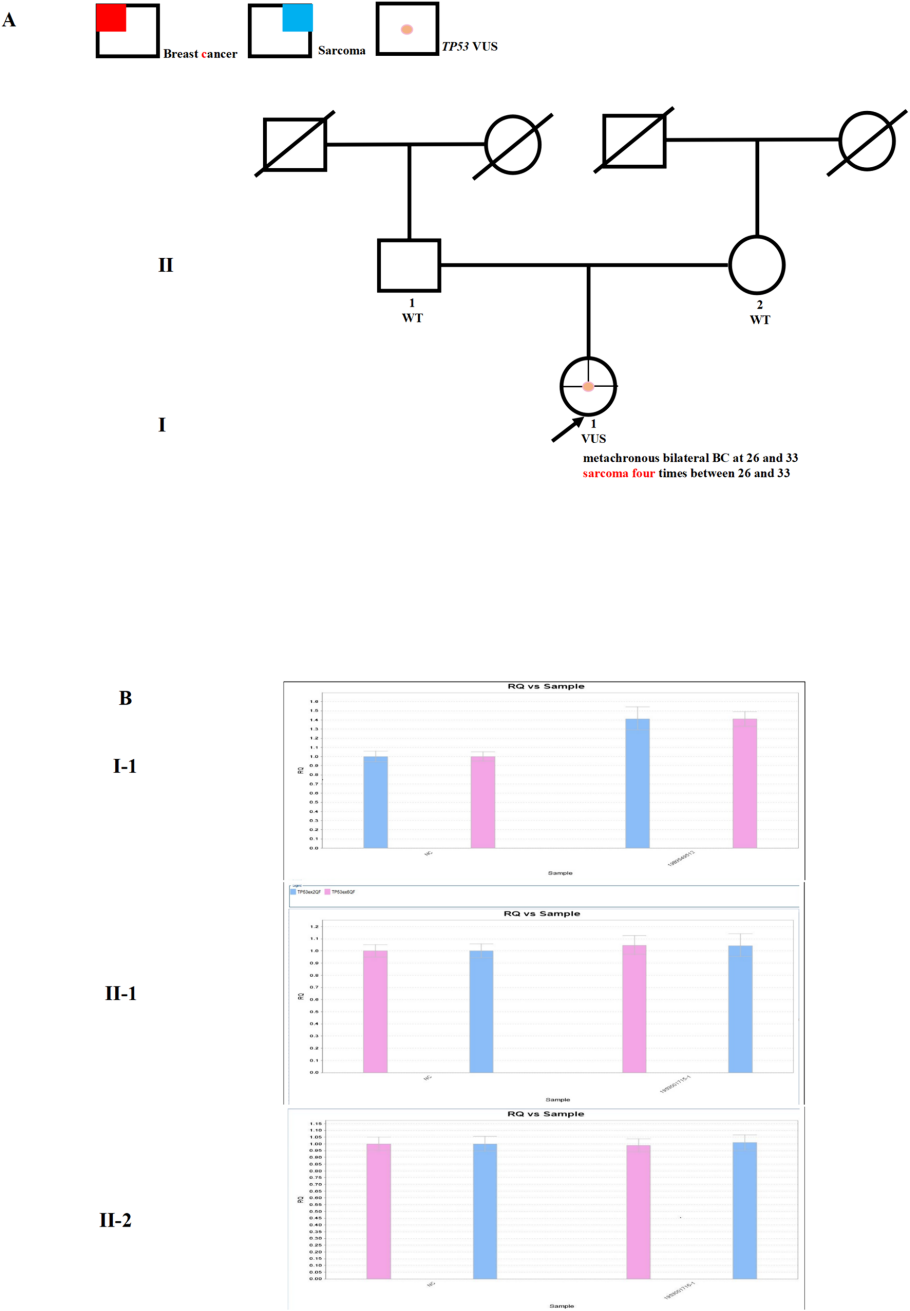
Germline *TP53* mutation identified in Case 7. **A** Pedigree of the family in Case 7. The proband is indicated with an arrow. The orange spot denotes a *TP53* variant of unknown significance (VUS). The red color denotes the manifestation of breast cancer (BC) and the blue color denotes–sarcoma. **B** Sanger sequencing (NM_000546, EX2_6 Dup) of the family showed that nobody harbored this variant. WT: wild-type. (Color figure online)

Case 7 had a specific *TP53* VUS, with clinical manifestations that seemed to harbour a deleterious *TP53* mutation. The woman was diagnosed with metachronous bilateral IDC at age 26 and 33. Immunohistochemical results revealed right TNBC and left breast cancer with ER-positive, PR-positive, and HER2-negative. Although she underwent mastectomy for both breasts, she still developed recurrent sarcoma on the chest wall four times from age 26 to 33. She had no family history suggestive of LFS (Fig. 5A). NGS was recommended, and a duplication mutation was detected (NM_000546: EX2_6 Dup). The mutation resulted in the duplication of exons 2–6 of the gene. However, functional studies on the clinical significance of this mutation have not yet been reported. Therefore, the mutation was considered to be of uncertain significance. However, due to both bilateral metachronous breast cancer and recurrent sarcoma, we believe that the mutation has potential pathogenic significance. Still, the same mutation was not detected in her parents via qPCR (Fig. 5B).

## Discussion

The present study revealed the underidentification of germline *TP53* mutations in patients with breast cancer, which was associated with inconsistency between diverse phenotypes and testing criteria. To the best of our knowledge, this is the first such study in the Chinese population. In particular, pedigree analysis was used to investigate the underlying cause of the inconsistency.

We adopted testing criteria aimed at high-penetrance breast cancer susceptibility genes from NCCN Guidelines (supplementary Table 1) [1]. Therefore, we aimed to investigate this high-risk population. Moreover, we added a new testing criterion, bilateral breast cancer, which is more common in both *TP53* carriers and *BRCA* carriers [19, 23]. We found that the rate of deleterious *TP53* mutation was higher (1.4%) in our high-risk population than in unselected populations (0.5%) [8]. Furthermore, the rate of *BRCA1/2* carriers (21.0%) in our study population was equal to that reported in patients with familial breast cancer (18.1%) [24, 25].

Previously, a *TP53* test was recommended for patients with breast cancer aged < 31 years [6, 26]. Although *TP53* mutation carriers seemed to develop breast cancer earlier than did *BRCA* mutation carriers and non-carriers in the present study, most of them (66.7%, 4/6) still developed breast cancer after 31 years. By contrast, only 33.3% (2/6) of *TP53* carriers had a family history suggestive of LFS (Table 2), which was also inconsistent with current guidelines [11]. Furthermore, bilateral breast cancer was more common in *TP53* carriers than in *BRCA* carriers, demonstrating that the previous *TP53* testing criteria may not be precisely suitable for Chinese patients with breast cancer. Therefore, a new set of criteria should be adopted in clinical practice, especially for Chinese patients, as it is necessary to identify as many patients with breast cancer with *TP*53 mutations as possible.

The latest 2021 guidelines recommend that patients at high risk undergo breast cancer susceptibility gene panel testing [1]. However, another study reported that it is rare for *TP53* carriers to lack a family history of cancer; therefore, it is unreasonable that *TP53* testing is offered to patients with early_onset breast cancer without a family history of cancer [27]. In contrast, in the present study, more Chinese *TP*53 carriers (4/6) lacked a family history suggestive of LFS. Likewise, in Case 4, the patient did not receive genetic testing for *TP53* screening even though she developed early onset breast cancer in the absence of a typical family history at initial diagnosis. Therefore, the age of onset may be more important than a typical family history as the testing criteria for identifying germline *TP53* mutations, especially when selecting surgical management. In agreement with our findings, Bakhuizen et al. reported that the majority (5/8) lacked the family history suggestive of LFS in a cohort of eight *TP53* carriers with breast cancer [26]. Rana HQ et al. also suggested that potential *TP*53 carriers can be identified in nonclassical LFS families even if phenotypes are not obvious [10]. We assumed that a family history suggestive of LFS would not be highly necessary as a testing criterion for identifying germline *TP53* mutations in patients with breast cancer, especially in the Chinese population.

Furthermore, we explored the potential cause of the inconsistency through pedigree analysis. We investigated the mutation status in the pedigrees of four *TP53* carriers and ultimately found that none of the family members harbored the same *TP53* mutation as did the probands. We assumed that the mutations might occur de novo [28]. This could explain why there was a lack of family history suggestive of LFS for *TP53* carriers. Renaux-Petel et al. estimated that the rate of de novo mutations in *TP53* reached 14% (48/336), nearly 20% of which occur during embryonic development [29]. According to Gonzalez et al., the frequency of de novo mutations in *TP53* might reach 20% [28]. However, a low VAF was still observed in our study. Due to the diverse capture and/or amplification efficiency during library construction, VAF can be distributed over a wide range, 30%–50%, for most situations or even lower in some cases. In case 1, the VAF had a distribution of 29.3% (Table 2), which can be considered reasonable. Conversely, a low VAF may also suggest constitutional mosaicism, as Renaux-Petel et al. reported mosaic mutations in 8 of 48 patients with de novo *TP53* mutations [29]. Notably, in Case 3, the aunt on the father’s side harbored the same mutation as did the proband (Fig. 3). We assumed that this may be due to a germline mosaic mutation in her father. Unfortunately, the patients refused further testing using other non-blood-derived DNA samples to address this possibility. The low VAF of the variants could have arisen from clonal hematopoiesis [30, 31]. Approximately 23 − 38% of low-VAF *TP53* variants would originate from clonal hematopoiesis [30]. Considering that de novo mutations/mosaicism may contribute to non-classic LFS families for *TP53* carriers, medical laboratories should ensure the detection of de novo/mosaic mutations. More importantly, variants might arise due to clonal hematopoiesis, which can confound germline *TP53* mutations and lead to erroneous conclusions in clinical practice [32].

We also found that *TP53* carriers were more likely to develop bilateral breast cancer than both *BRCA* carriers and non-carriers (Table 2), similar to previous findings [8, 33]. Considering the radiation exposure from post-BCS radiotherapy in pathogenic/likely pathogenic *TP53* variant carriers, bilateral mastectomy should be favored in risk reduction of sarcoma development [13]. Furthermore, *TP*53 mutation is an independent unfavorable prognostic factor for patients with breast cancer [8, 33]. In the present study, patients with *TP*53 germline mutations were more likely to develop two or even three primary cancers. Since multigene assay testing has become routine in China, the testing criteria for *TP*53 should be appropriately expanded to avoid omission.

Intriguingly, Case 7 also carried a *TP53* duplication variant (EX2_6 Dup), which was defined as ‘uncertain significance,’ and her phenotype (early onset bilateral breast cancer and recurrent chest wall sarcomas) was highly consistent with that of *TP53* carriers, which suggests the variant is associated with cancer predisposition. Unfortunately, our study showed that functional verification is required for the reclassification of a VUS, which is very difficult to achieve. [34] Considering the specificity of the therapy for patients with breast cancer with deleterious *TP53* mutations, we treated her clinically as a *TP53* carrier. This variant warrants further research and validation to establish whether we can consider patients with breast cancer with specific *TP53* VUSs as *TP53* carriers in clinical practice.

The present study had several limitations. First, the sample size of *TP53* carriers was relatively small. Therefore, we will continue to expand the sample size to further evaluate the relevant features. Second, we determined the *TP53* germline mutation status in a high-risk population; therefore, clinicopathological characteristics should be discussed in a high-risk population. Furthermore, we did not investigate the pedigrees of all *TP53* carriers; therefore, the possible contribution of de novo and mosaic *TP53* mutations might be greater than expected. Moreover, other non-blood-derived DNA samples need to be used to confirm de novo/mosaic mutations. The possible variants arising due to clonal hematopoiesis may need attention. Finally, LGR of gene *TP53* may not be detected or verified using MGPT and Sanger sequencing, which may result in underestimation.

## Conclusions

There are distinct features of *TP53* carriers in Chinese breast cancer patients. They can develop breast cancer after age 31, and bilateral breast cancer is also common. Chinese patients with breast cancer with *TP53* mutations usually originate from non-classic LFS families. Considering the inconsistency between the diverse phenotypes and testing criteria for germline *TP53* mutations, the contribution of de novo and mosaic *TP53* mutations might be the potential cause. However, the variants could also arise owing to clonal hematopoiesis. Nonetheless, the currently used testing criteria for germline *TP53* carriers might not be entirely appropriate for Chinese patients with breast cancer. More attention should be paid to age and individual tumor history when considering *TP53* gene testing. To avoid the underidentification of germline *TP53* mutations in such patients, we propose a new testing criterion (paragraph 2.2). Owing to the difficulty of upgrading *TP53* VUSs and treatment specificity, it might be preferable to consider a Chinese breast cancer patient with a specific *TP53* VUS as a *TP53* carrier in clinical practice, if the clinical manifestation of the patient conforms to that of a deleterious *TP53* mutation.

### Supplementary Information

Below is the link to the electronic supplementary material.

Supplementary file1 (DOCX 26 KB)

Supplementary file2 (DOCX 26 KB)

## Data Availability

Data sharing is not applicable.

## Abbreviations

BCS: Breast-conserving surgery
CIN: Cervical intraepithelial neoplasia
ER: Estrogen receptor
gDNA: Genomic DNA
IDC: Invasive ductal carcinoma
LFS: Li-Fraumeni syndrome
MGPT: Multiplex genetic panel testing
P/LP: Pathogenic or likely pathogenic
PR: Progesterone receptor
RT: Radiation therapy
SLNB: Sentinel lymph node biopsy
STS: Soft-tissue sarcomas
TNBC: Triple-negative breast cancer
VAF: Variant allele frequency

## References

[1] Daly MB, Pal T, Berry MP, Buys SS, Dickson P, Domchek SM, Elkhanany A, Friedman S, Goggins M, Hutton ML, Karlan BY (2021). Genetic/familial high-risk assessment: breast, ovarian, and pancreatic, version 2.2021, NCCN clinical practice guidelines in oncology. J Natl Compr Canc Netw.

[2] Guha T, Malkin D (2017). Inherited TP53 mutations and the Li-Fraumeni syndrome. Cold Spring Harb Perspect Med.

[3] de Andrade KC, Khincha PP, Hatton JN, Frone MN, Wegman-Ostrosky T, Mai PL, Best AF, Savage SA (2021). Cancer incidence, patterns, and genotype-phenotype associations in individuals with pathogenic or likely pathogenic germline TP53 variants: an observational cohort study. Lancet Oncol.

[4] Xia C, Dong X, Li H, Cao M, Sun D, He S, Yang F, Yan X, Zhang S, Li N, Chen W (2022). Cancer statistics in China and United States, 2022: profiles, trends, and determinants. Chin Med J (Engl).

[5] Tuna M, Ju Z, Yoshihara K, Amos CI, Tanyi JL, Mills GB (2020). Clinical relevance of TP53 hotspot mutations in high-grade serous ovarian cancers. Br J Cancer.

[6] McCuaig JM, Armel SR, Novokmet A, Ginsburg OM, Demsky R, Narod SA, Malkin D (2012). Routine TP53 testing for breast cancer under age 30: ready for prime time?. Fam Cancer.

[7] Bougeard G, Renaux-Petel M, Flaman JM, Charbonnier C, Fermey P, Belotti M, Gauthier-Villars M, Stoppa-Lyonnet D, Consolino E, Brugières L, Caron O, Benusiglio PR, Bressac-de Paillerets B, Bonadona V, Bonaïti-Pellié C, Tinat J, Baert-Desurmont S, Frebourg T (2015). Revisiting Li-Fraumeni syndrome from TP53 mutation carriers. J Clin Oncol.

[8] Sheng S, Xu Y, Guo Y, Yao L, Hu L, Ouyang T, Li J, Wang T, Fan Z, Fan T, Lin B, Xie Y (2020). Prevalence and clinical impact of TP53 germline mutations in Chinese women with breast cancer. Int J Cancer.

[9] Packwood K, Martland G, Sommerlad M, Shaw E, Moutasim K, Thomas G, Bateman AC, Jones L, Haywood L, Evans DG, Birch JM, Alsalmi OA, Henderson A, Poplawski N, Eccles DM (2019). Breast cancer in patients with germline TP53 pathogenic variants have typical tumour characteristics: the Cohort study of TP53 carrier early onset breast cancer (COPE study). J Pathol Clin Res.

[10] Rana HQ, Gelman R, LaDuca H, McFarland R, Dalton E, Thompson J, Speare V, Dolinsky JS, Chao EC, Garber JE (2018). Differences in TP53 mutation carrier phenotypes emerge from panel-based testing. J Natl Cancer Inst.

[11] Daly MB, Pilarski R, Berry M, Buys SS, Farmer M, Friedman S, Garber JE, Kauff ND, Khan S, Klein C, Kohlmann W, Kurian A, Litton JK, Madlensky L, Merajver SD, Offit K, Pal T, Reiser G, Shannon KM, Swisher E, Vinayak S, Voian NC, Weitzel JN, Wick MJ, Wiesner GL, Dwyer M, Darlow S (2017). NCCN guidelines insights: genetic/familial high-risk assessment: breast and ovarian, version 2.2017. J Natl Compr Canc Netw.

[12] Tung NM, Boughey JC, Pierce LJ, Robson ME, Bedrosian I, Dietz JR, Dragun A, Gelpi JB, Hofstatter EW, Isaacs CJ, Jatoi I, Kennedy E, Litton JK, Mayr NA, Qamar RD, Trombetta MG, Harvey BE, Somerfield MR, Zakalik D (2020). Management of hereditary breast cancer: American society of clinical oncology, American society for radiation oncology, and society of surgical oncology guideline. J Clin Oncol.

[13] Heymann S, Delaloge S, Rahal A, Caron O, Frebourg T, Barreau L, Pachet C, Mathieu MC, Marsiglia H, Bourgier C (2010). Radio-induced malignancies after breast cancer postoperative radiotherapy in patients with Li-Fraumeni syndrome. Radiat Oncol.

[14] Fisher B, Anderson S, Bryant J, Margolese RG, Deutsch M, Fisher ER, Jeong JH, Wolmark N (2002). Twenty-year follow-up of a randomized trial comparing total mastectomy, lumpectomy, and lumpectomy plus irradiation for the treatment of invasive breast cancer. N Engl J Med.

[15] Guan Y, Hu H, Peng Y, Gong Y, Yi Y, Shao L, Liu T, Li G, Wang R, Dai P, Bignon YJ, Xiao Z, Yang L, Mu F, Xiao L, Xie Z, Yan W, Xu N, Zhou D, Yi X (2015). Detection of inherited mutations for hereditary cancer using target enrichment and next generation sequencing. Fam Cancer.

[16] Huang X, Cai XY, Liu JQ, Hao WW, Zhou YD, Wang X, Xu Y, Chen C, Lin Y, Wang CJ, Song Y, Sun Q (2020). Breast-conserving therapy is safe both within BRCA1/2 mutation carriers and noncarriers with breast cancer in the Chinese population. Gland Surg.

[17] Sun S, Liu Y, Eisfeld AK, Zhen F, Jin S, Gao W, Yu T, Chen L, Wang W, Chen W, Yuan M, Chen R, He K, Guo R (2019). Identification of germline mismatch repair gene mutations in lung cancer patients with paired tumor-normal next generation sequencing: a retrospective study. Front Oncol.

[18] Fortuno C, Lee K, Olivier M, Pesaran T, Mai PL, de Andrade KC, Attardi LD, Crowley S, Evans DG, Feng BJ, Foreman AKM, Frone MN, Huether R, James PA, McGoldrick K, Mester J, Seifert BA, Slavin TP, Witkowski L, Zhang L, Plon SE, Spurdle AB, Savage SA, ClinGen TP53 Variant Curation Expert Panel (2021). Specifications of the ACMG/AMP variant interpretation guidelines for germline TP53 variants. Hum Mutat.

[19] Richards S, Aziz N, Bale S, Bick D, Das S, Gastier-Foster J, Grody WW, Hegde M, Lyon E, Spector E, Voelkerding K, Rehm HL, ACMG Laboratory Quality Assurance Committee (2015). Standards and guidelines for the interpretation of sequence variants: a joint consensus recommendation of the American college of medical genetics and genomics and the association for molecular pathology. Genet Med.

[20] Huang X, Shao D, Wu H, Zhu C, Guo D, Zhou Y, Chen C, Lin Y, Lu T, Zhao B, Wang C, Sun Q (2020). Genomic profiling comparison of germline BRCA and non-BRCA carriers reveals CCNE1 amplification as a risk factor for non-BRCA carriers in patients with triple-negative breast cancer. Front Oncol.

[21] Braxton DR, Zhang R, Morrissette JD, Loaiza-Bonilla A, Furth EE (2016). Clinicopathogenomic analysis of mismatch repair proficient colorectal adenocarcinoma uncovers novel prognostic subgroups with differing patterns of genetic evolution. Int J Cancer.

[22] Gonin-Laurent N, Gibaud A, Huygue M, Lefèvre SH, Le Bras M, Chauveinc L, Sastre-Garau X, Doz F, Lumbroso L, Chevillard S, Malfoy B (2006). Specific TP53 mutation pattern in radiation-induced sarcomas. Carcinogenesis.

[23] Mruthyunjayappa S, Zhang K, Zhang L, Eltoum IA, Siegal GP, Wei S (2019). Synchronous and metachronous bilateral breast cancer: clinicopathologic characteristics and prognostic outcomes. Hum Pathol.

[24] Sun J, Meng H, Yao L, Lv M, Bai J, Zhang J, Wang L, Ouyang T, Li J, Wang T, Fan Z, Fan T, Lin B, Xie Y (2017). Germline mutations in cancer susceptibility genes in a large series of unselected breast cancer patients. Clin Cancer Res.

[25] Bakhuizen JJ, Hogervorst FB, Velthuizen ME, Ruijs MW, van Engelen K, van Os TA, Gille JJ, Collée M, van den Ouweland AM, van Asperen CJ, Kets CM, Mensenkamp AR, Leter EM, Blok MJ, de Jong MM, Ausems MG (2019). TP53 germline mutation testing in early-onset breast cancer: findings from a nationwide cohort. Fam Cancer.

[26] Ginsburg OM, Akbari MR, Aziz Z, Young R, Lynch H, Ghadirian P, Robidoux A, Londono J, Vasquez G, Gomes M, Costa MM, Dimitrakakis C, Gutierrez G, Pilarski R, Royer R, Narod SA (2009). The prevalence of germ-line TP53 mutations in women diagnosed with breast cancer before age 30. Fam Cancer.

[27] Gonzalez KD, Buzin CH, Noltner KA, Gu D, Li W, Malkin D, Sommer SS (2009). High frequency of de novo mutations in Li-Fraumeni syndrome. J Med Genet.

[28] Renaux-Petel M, Charbonnier F, Théry JC, Fermey P, Lienard G, Bou J, Coutant S, Vezain M, Kasper E, Fourneaux S, Manase S, Blanluet M, Leheup B, Mansuy L, Champigneulle J, Chappé C, Longy M, Sévenet N, Paillerets BB, Guerrini-Rousseau L, Brugières L, Caron O, Sabourin JC, Tournier I, Baert-Desurmont S, Frébourg T, Bougeard G (2018). Contribution of de novo and mosaic TP53 mutations to Li-Fraumeni syndrome. J Med Genet.

[29] Schwartz AN, Hyman SR, Stokes SM, Castillo D, Tung NM, Weitzel JN, Rana HQ, Garber JE (2021). Evaluation of TP53 variants detected on peripheral blood or saliva testing: discerning germline from somatic TP53 variants. JCO Precis Oncol.

[30] Batalini F, Peacock EG, Stobie L, Robertson A, Garber J, Weitzel JN, Tung NM (2019). Li-Fraumeni syndrome: not a straightforward diagnosis anymore-the interpretation of pathogenic variants of low allele frequency and the differences between germline PVs, mosaicism, and clonal hematopoiesis. Breast Cancer Res.

[31] Weitzel JN, Chao EC, Nehoray B, Van Tongeren LR, LaDuca H, Blazer KR, Slavin T, Facmg DABMD, Pesaran T, Rybak C, Solomon I, Niell-Swiller M, Dolinsky JS, Castillo D, Elliott A, Gau CL, Speare V, Jasperson K (2018). Somatic TP53 variants frequently confound germ-line testing results. Genet Med.

[32] Escudeiro C, Pinto C, Vieira J, Peixoto A, Pinto P, Pinheiro M, Santos C, Guerra J, Lisboa S, Santos R, Silva J, Leal C, Coimbra N, Lopes P, Ferreira M, Sousa AB, Teixeira MR (2021). The role of TP53 pathogenic variants in early-onset HER2-positive breast cancer. Fam Cancer.

[33] Mersch J, Brown N, Pirzadeh-Miller S, Mundt E, Cox HC, Brown K, Aston M, Esterling L, Manley S, Ross T (2018). Prevalence of variant reclassification following hereditary cancer genetic testing. JAMA.

