# Supplementary material for: Pedigree analysis exploring the inconsistency between diverse phenotypes and testing criteria for germline TP53 mutations in Chinese women with breast cancer
Source: Breast Cancer Res Treat. 2024 Jun 15;206(3):653–66. doi: 10.1007/s10549-024-07341-7 (PMC11208215; doi:10.1007/s10549-024-07341-7)
Supplement: Supplementary file 1 — Supplementary file1 (DOCX 26 KB) [file 10549_2024_7341_MOESM1_ESM.docx]

**Supplementary Table 1**

| **Testing criteria aimed at high-penetrance breast cancer susceptibility genes**^†^ | |
| --- | --- |
| 1 | Breast cancer diagnosed at age ≤45 y |
| 2 | Diagnosed at age ≤60 y with triple-negative breast cancer |
| 3 | Diagnosed at age 46–50 y with:  ◊ Unknown or limited family history; or  ◊ A second breast cancer diagnosed at any age; or  ◊ ≥1 close blood relative with breast, ovarian, pancreatic, or prostate cancer at any age |
| 4 | Diagnosed at any age with:  ◊ Ashkenazi Jewish ancestry; or  ◊ ≥1 close blood relative with breast cancer at age ≤50 y or ovarian, pancreatic, metastatic, intraductal/cribriform histology, or high- or very-high risk group prostate cancer at any age; or  ◊ ≥3 total diagnoses of breast cancer in the patient and/or close blood relatives |
| 5 | Diagnosed at any age with male breast cancer |

^†^ From Genetic/Familial High-Risk Assessment: Breast, Ovarian, and Pancreatic, Version 2.2021, NCCN Clinical Practice Guidelines in Oncology.
