# Supplementary material for: Pedigree analysis exploring the inconsistency between diverse phenotypes and testing criteria for germline TP53 mutations in Chinese women with breast cancer
Source: Breast Cancer Res Treat. 2024 Jun 15;206(3):653–66. doi: 10.1007/s10549-024-07341-7 (PMC11208215; doi:10.1007/s10549-024-07341-7)
Supplement: Supplementary file 2 — Supplementary file2 (DOCX 26 KB) [file 10549_2024_7341_MOESM2_ESM.docx]

**Supplementary Table 2.**

| **Test panel** | |
| --- | --- |
| Genes | Scope of the test |
| *BRCA1, BRCA2, CHEK2, PALB2, BRIP1, TP53, PTEN, STK11, CDH1, ATM, BARD1, MLH1, MRE11A, MRE11A, MSH2, MSH6, MUTYH, NBN, PMS1, PMS2, RAD50, RAD51C, RAD51D, NF1, EPCAM,* and *SMARCA4* | Exon and adjacent ±10 bp intron regions |
